# Safety and efficacy of sintilimab combined with oxaliplatin/capecitabine as first-line treatment in patients with locally advanced or metastatic gastric/gastroesophageal junction adenocarcinoma in a phase Ib clinical trial

**DOI:** 10.1186/s12885-020-07251-z

**Published:** 2020-08-14

**Authors:** Haiping Jiang, Yulong Zheng, Jiong Qian, Chenyu Mao, Xin Xu, Ning Li, Cheng Xiao, Huan Wang, Lisong Teng, Hui Zhou, Shuyan Wang, Donglei Zhu, Bo Peng, Lin Shen, Nong Xu

**Affiliations:** 1 Department of Medical Oncology, The First Affiliated Hospital, School of Medicine, Zhejiang University, No. 79, 86 Qingchun Road, Hangzhou, 310003 China; 2 Department of Surgical Oncology, The First Affiliated Hospital, School of Medicine, Zhejiang University, Hangzhou, China; 3 Department of Medical Science and Strategy Oncology, Innovent Biologics, Inc, Suzhou, China; 4 Department of Translational Medicine, Innovent Biologics, Inc, Suzhou, China; 5 Department of Medical Oncology, Beijing Cancer Hospital, Beijing, China

**Keywords:** Sintilimab, Capecitabine, Oxaliplatin, Gastric/gastroesophageal junction adenocarcinoma, Tumor mutation burden

## Abstract

**Background:**

Sintilimab blocks the interaction between programmed death-1 (PD-1) and its ligands. The safety and efficacy of sintilimab combined with oxaliplatin/capecitabine (CapeOx) as first-line treatment were evaluated in patients with gastric (G)/gastroesophageal junction (GEJ) adenocarcinoma in a phase Ib clinical trial.

**Methods:**

Patients with locally advanced or metastatic G/GEJ adenocarcinoma without previous systemic treatment were enrolled as one cohort of a multi-cohort study. Sintilimab was administered at a dose of 200 mg intravenously (IV) in combination with CapeOx (1000 mg/m^2^ capecitabine orally, bid, D1–14 and 130 mg/m^2^ oxaliplatin IV, D1) every 21 days for up to 6 cycles. After combination treatment, patients continued to receive sintilimab (200 mg) at 3 weekly intervals as maintenance therapy until progressive disease (PD), unacceptable toxicity, withdrawal of informed consent, or for up to 24 months. Adverse events (AEs) were monitored to assess safety in terms of their frequency, intensity and causality. The efficacy endpoints included the objective response rate (ORR), disease control rate (DCR), progression-free survival (PFS) and overall survival (OS). Tumor mutation burden (TMB) was evaluated for its association with clinical response.

**Results:**

A total of 20 patients were enrolled and received sintilimab plus CapeOx. All patients reported treatment-related AEs (TRAEs). Grade 3–4 TRAEs were found in 11 (55.0%) patients. Seventeen patients obtained partial response and the ORR was 85.0% (95% CI: 62.1–96.8%). Three (15.0%) had stable disease and DCR was 100.0% (95% CI: 83.2–100.0%). As data cutoff of May 1, 2019, the median follow-up was 7.8 months. The median PFS was 7.5 months (95% CI: 6.2–9.4) and median OS had not been reached. The OS rates at 6 months and 12 months were 100.0 and 68.0%. No association was observed between TMB and efficacy.

**Conclusions:**

Sintilimab combined with CapeOx as first-line treatment demonstrated acceptable safety and promising efficacy.

**Trial registration:**

ClinicalTrials.gov, NCT02937116. Registered 8 October 2016.

## Background

The fifth most commonly diagnosed cancer worldwide is gastric cancer (GC), accounting for about 33% of cancer-related deaths globally and the third most common cancer in China with almost half of worldwide new GC cases occurring in China annually [1, 2]. The standard treatments exhibit regional differences among western countries, Japan/Korea and China, which are considered to be associated with different screening and early detection methods as well as different biological behaviors, disease characteristics and ethnicity [3–5].

Surgical resection is the only radical therapy for gastric/gastroesophageal junction (G/GEJ) cancer. However, systemic chemotherapy is an alternative main therapy for G/GEJ cancer because of the high relapse rate after post-resection surgery and for the many patients diagnosed at an advanced-stage. For advanced G/GEJ cancer, first-line treatment mainly involves platinum-based chemotherapy using a combination of two or three drugs (trastuzumab is given to patients whose tumor is human epidermal growth factor receptor-2 (HER2) positive), but the overall survival (OS) is disappointing, since the maximum OS time has been reported to be 13.8 months [6–10]. Any potential novel drug that will increase patient survival times is urgently needed, in particular for first-line treatment.

Immune checkpoint inhibitor treatment is a new approach for tumor immunotherapy [11, 12]. The treatment diminishes the immune system tolerance to tumor cells and improves the effective identification and eradication of tumor cells by blocking T cell inhibition [13]. The programmed death-1 (PD-1) antibody specifically binds to PD-1, thereby inhibiting apoptosis of antigen-specific T cells and thus reducing regulatory T cell (Treg) apoptosis by inhibiting the activation of PD-L1 [14, 15].

The efficacy of anti-PD-1 antibodies monotherapy in patients who had prior chemotherapy for advanced GC has been demonstrated and supported by several trials. In the KEYNOTE-012 and KEYNOTE-059 trials, pembrolizumab monotherapy showed objective response rates (ORR) of 22% (*n* = 36) [16] and 15.5% (*n* = 148) [17], respectively in PD-L1 positive advanced GC patients after at least two prior systemic therapies. Based on such results, the Food and Drug Administration approved pembrolizumab for third-line treatment of patients with recurrent or advanced GC. In the ATTRACTION-2 study, nivolumab monotherapy improved OS from 4.1 to 5.3 months (hazard ratio 0.63, 95% CI: 0.51–0.78; *P* < 0.000), compared with a placebo in advanced GC that was refractory or intolerant to previous treatment regimens [18].

However, between 30 and 60% of patients exhibit no response to PD-1 blockade, which is considered to be associated with T cell exclusion or exhaustion or inadequate T cell trafficking and many immunosuppressive factors accumulate in the tumor microenvironment [19]. New therapy regimens that improve the response and long-term efficacy are desperately needed. The efficacy of anti-PD-1 therapy in combination with chemotherapy has been confirmed in non-small-cell lung cancer [20, 21]. In addition to direct tumor killing, conventional cytotoxic chemotherapy has demonstrated immunoregulatory properties by enhancing tumor antigenicity, disturbing immune suppressive pathways, inducing immunogenic cell death, and increasing effector T-cell reactions [22]. It is safe to hypothesize that anti-PD-1 antibodies in combination with chemotherapy may further improve the clinical outcomes of patients with advanced GC. Sintilimab is a highly selective, monoclonal IgG4 antibody that inhibits interactions between PD-1 and its ligands, with strong anti-tumor response [23]. A phase 1a study for dose escalation, has demonstrated the tolerance and pharmacological activity of sintilimab in patients with advanced-stage solid tumors, but there is limited evidence for the efficacy of antibodies against PD-1 plus chemotherapy in Chinese G/GEJ adenocarcinoma patients. Thus, the present trial was conducted to investigate the safety and efficacy of sintilimab combined with CapeOx as first-line therapy for a cohort of patients with G/GEJ adenocarcinoma.

## Methods

### Study design and patients

The present study was an open label, multicenter, phase Ib study to evaluate the safety and efficacy of sintilimab in 6 cohorts of patients with solid tumors. Patients (age range 18–70 years) with cytologically or histologically confirmed unresectable G/GEJ adenocarcinoma were enrolled in the G/GEJ cohort. Tumor, nodes and metastases (TNM) staging has been evaluated according to the Union for International Cancer Control (UICC) TNM classification 8th edition [24]. The patients had received no previous systemic treatment for advanced disease or had disease progression (PD) more than 6 months after systemic adjuvant therapy. Other major inclusion criteria were: at least one measurable lesion as defined by the Response Evaluation Criteria in Solid Tumor (RECIST version 1.1) criteria; score 0 or 1 for Eastern Tumor Collaborative Group Performance Status (ECOG-PS); adequate organ and bone marrow functions and life expectancy ≥12 weeks. Patients with amplification or overexpression of the HER2 gene were excluded from the trial. Appendix 1 contains a complete list of all inclusion and exclusion criteria.

The institutional review boards of all centers approved the protocols and the study was carried out in strict accordance with the declaration of Helsinki principles; all participating patients signed consent forms before taking part.

### Procedures

According to NCCN guideline, the preferred first-line chemotherapy regimens for advanced gastric cancer are fluorouracil or capecitabine combined with cisplation or oxaliplatin [25]. However, the results from the REAL-2 study [26] revealed significant clinical benefit of the oxaliplatin/capecitabine (CapeOx) regimen which led to the longest OS time of 11.2 months, compared with other regimens. Oxaliplatin produces less renal toxicity, there is no requirement for hydration and it has a lower emetic potential compared to cisplatin, while capecitabine has no requirement for continuous intravenous (IV) infusion and is administered orally, which should ensure an improved quality of life for patients in their homes. Therefore, a CapeOx regimen has been chosen. During the combination phase, enrolled patients were given sintilimab in combination with CapeOx for up to 6 cycles (every 3 weeks). Each cycle consisted of intravenous sintilimab (200 mg) plus oxaliplatin (130 mg/m^2^) on day 1 and capecitabine (1000 mg/m^2^ twice daily orally) from day 1 to day 14. After combination treatment, patients without PD continued to receive sintilimab (200 mg) at 3 weekly intervals as maintenance therapy until PD, unacceptable toxic effects, withdrawal of informed consent, or for up to 24 months.

### Study assessments

Adverse events (AEs) were monitored for 90 days after the last administration of a treatment dose. Responses were assessed by computed tomography (CT) or magnetic resonance imaging (MRI), every 9 weeks until PD, new treatment initiation, withdrawal of informed consent, or death.

### Endpoints

Safety was assessed as collected AEs according to their type, frequency, causality and severity grading defined by the National Cancer Institute Common Terminology Criteria (CTCAE) ver. 4.03. The efficacy endpoints were the ORR, disease control rate (DCR), time to response (TTR), duration of response (DOR), progression free survival (PFS) and OS. Efficacy was determined by an investigator according to RECIST v1.1 guidelines.

Exploratory endpoints were to evaluate the correlation of tumor mutation burden (TMB) with clinical efficacy.

### Tumor mutation burden analysis

The tumor biopsies and blood samples were collected at baseline. DNA sequences were extracted from biopsies of tumors with matched blood samples and submitted for next generation sequencing using a designed 1622-gene panel (Genecast, Beijing, China). TMB was determined by analysis of the quantity of somatic mutations per megabase (Mb). Median TMB was used as a cut-off to define a tumor as high-TMB (H-TMB) and low-TMB (L-TMB).

### Statistical analysis

All patients who received at least one study treatment were included in the safety and efficacy analyses. AEs were coded following the Medical Dictionary for Regulatory Activity and tabulated by system organ class and preferred terms. Causality between AEs and the study treatment was assessed by the investigator. ORR was calculated as the proportion of patients who had achieved a complete response (CR) or partial response (PR) and the 95% CIs were evaluated by the binomial distribution. DCR was calculated as the proportion of patients who obtained PR, CR and stable disease (SD) and data are presented with the 95% CIs. Median DOR, TTR, PFS, OS and the PFS and OS rates at 6 and 12 months were determined using the Kaplan-Meier methodology. Fisher’s test was used to compare the ORRs between patients with H-TMB and L-TMB.

## Results

**Fig. 1:**
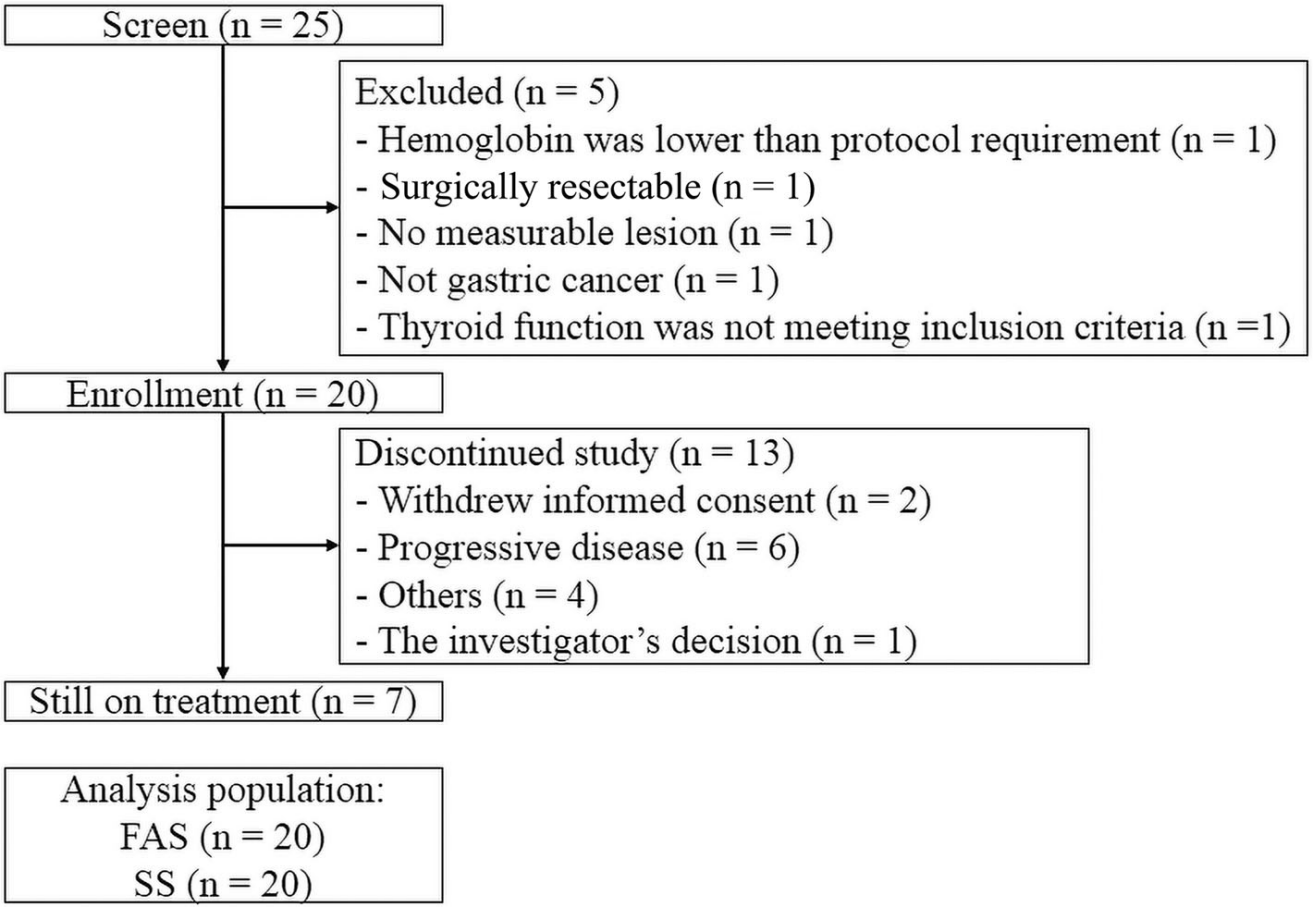
Flowchart of the study

From 26 Dec, 2017 to 17 Oct 2018, 25 patients were screened and 20 were enrolled in the G/GEJ adenocarcinoma cohort (Fig. 1). The median interval between initial diagnosis and screening was 14 days (range 7–611). Most patients (80.0%) had metastatic disease status and 11 (55.0%) had ECOG scores of 1 (Table 1). The TNM stage summary is shown in Table 1 and the staging of each patient in Supplementary Table 1.

**Table 1:** Demographics and disease characteristics

	All patients (*N* = 20)
Age (median, range) in years	59.8 (36.9 to 69.3)
Gender (n, %)	
Male	18 (90.0)
Female	2 (10.0)
ECOG PS (n, %)	
0	9 (45.0)
1	11 (55.0)
Time since initial diagnosis (median, range) in days	14 (7–611)
Disease status (n, %)	
Locally advanced	4 (20.0)
Metastatic	16 (80.0)
Location of the primary tumor (n, %)	
Upper	7 (35.0)
Middle	6 (30.0)
Lower	7 (35.0)
TNM staging (n, %)	
T3	3 (15.0)
T4	11 (55.0)
Tx	6 (30.0)
M0	4 (20.0)
M1	16 (80.0)
N1	1 (5.0)
N2	3 (15.0)
N3	6 (30.0)
Nx	10 (50.0)
Histology (n, %)	
Poorly differentiated adenocarcinoma	11 (55.0)
Moderately differentiated adenocarcinoma	5 (25.0)
Unknown differentiated adenocarcinoma	4 (20.0)

*ECOG* Eastern Cooperative Oncology Group, *T* tumor, *N* node, *M* metastasis

At data cutoff on May 1, 2019, the median follow-up time was 7.8 months (range 6.2–12.3). The median treatment duration was 6.2 months (range 2.1–10.4). All patients received more than 4 cycles of treatment, with the median doses of received sintilimab being 9.5 (range 4–16).

### Safety

All of the 20 patients reported at least one treatment-related adverse event (TRAE), and the most common TRAE was platelet count decreased (*n* = 16, 80.0%). Grade 3 or 4 treatment-related AEs (TRAEs) occurred in 11 (55.0%) patients, the most common also being a platelet count decreased (*n* = 9, 45.0%) (Table 2). No TRAE was fatal and 1 patient discontinued the treatment due to treatment-related Grade 3 hepatic function abnormal. Sintilimab-related AEs occurred in 17 (85.0%) patients. Grade 3–4 sintilimab-related AEs occurred in 5 (25.0%) patients, the most common being platelet count decreased (*n* = 3, 15.0%) (Supplementary Table 2). Chemotherapy-related AEs were found in all patients. Grade 3–4 chemotherapy-induced AEs were found in 11 patients (55.0%), the most common being platelet count decreased (*n* = 9, 45.0%) (Supplementary Table 3). Five patients reported treatment-related serious adverse events: platelet count decreased (*n* = 4), abnormal hepatic function (*n* = 1), hypothyroidism (n = 1), pneumonitis (n = 1) and autoimmune colitis (n = 1).

**Table 2:** Treatment-related adverse events (TRAEs)

	All grade n (%)	Grade 3–4 n (%)
**All TRAEs (n)**	**20 (100.0)**	**11 (55.0)**
Platelet count decreased	16 (80.0)	9 (45.0)
White blood cell count decreased	10 (50.0)	0 (0.0)
Neutrophil count decreased	10 (50.0)	2 (10.0)
Hypothyroidism	6 (30.0)	0 (0.0)
Rash	5 (25.0)	0 (0.0)
Alanine aminotransferase increased	5 (25.0)	0 (0.0)
Aspartate aminotransferase increased	4 (20.0)	0 (0.0)
Anemia	4 (20.0)	0 (0.0)
Hepatic function abnormal	3 (15.0)	1 (5.0)
Vomiting	3 (15.0)	0 (0.0)
Nausea	2 (10.0)	0 (0.0)
Hyperchlorhydria	2 (10.0)	0 (0.0)
Thyroid function test abnormal	2 (10.0)	0 (0.0)
Hypokalemia	2 (10.0)	1 (5.0)
Hypesthesia	2 (10.0)	0 (0.0)
Pyrexia	2 (10.0)	0 (0.0)
Proteinuria	2 (10.0)	0 (0.0)
γ-glutamyl transferase increased	1 (5.0)	1 (5.0)
Diarrhea	1 (5.0)	1 (5.0)
Autoimmune colitis	1 (5.0)	1 (5.0)
Pneumonitis	1 (5.0)	1 (5.0)

Listed are any grade TRAE found in ≥10% patients, and all grade 3–4 TRAEs

### Efficacy

**Fig. 2:**
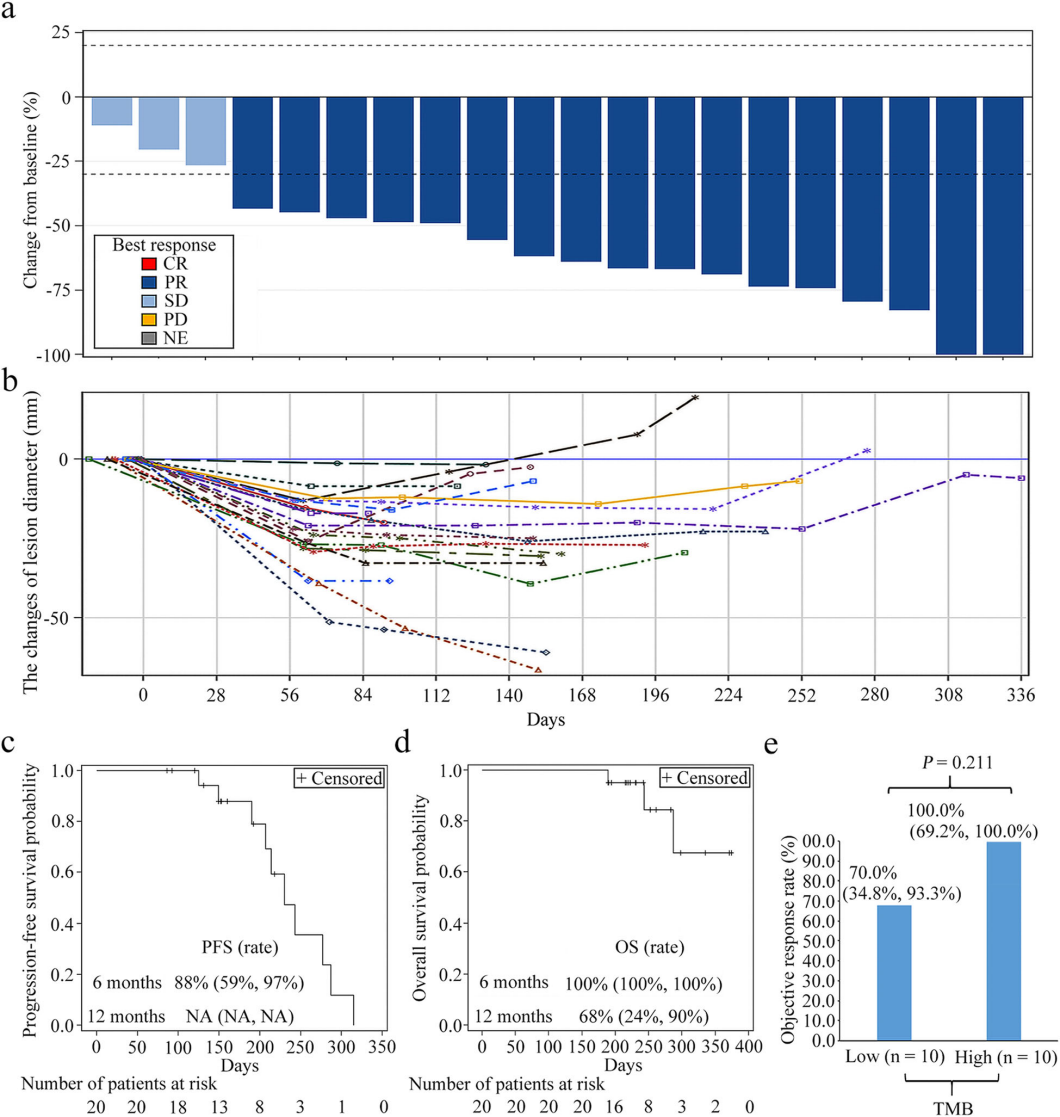
Evaluation of efficacy and tumor responses. **a**, Maximum change in tumor size from baseline. Seventeen of 20 patients obtained PR based on the percentage changes of the sum of the maximum diameter of the tumor lesion (range − 28% to − 100%); **b**, the change of lesion diameters over time from baseline; each line represents the changes in one patient; **c**, PFS Kaplan-Meier curve; d, OS Kaplan-Meier curve; e, the objective response rate in low and high TMB groups. OS, overall survival; PFS, progression-free survival; PR partial response; SD, stable disease; TMB, tumor mutation burden

All 20 patients experienced a decrease in the sum of their target lesions (Fig. 2a) and in the majority the lesions kept smaller than at baseline (Fig. 2b). The median TTR was 2.1 months (95% CI: 2.0–2.1) and the median DOR was 5.9 months (95% CI: 4.8–7.2). According to the best tumor response following RECIST 1.1 guidelines, 17 patients reached a PR (85.0% (95% CI: 62.1–96.8%)) and 15 (75.0%) patients obtained a confirmed objective response i.e. by two continuous PRs at intervals of 4 weeks. In addition, 3 patients had SD and DCR was 100.0% (95% CI: 83.2–100.0%) (Table 3).

**Table 3:** Efficacy evaluation of sintilimab

Efficacy evaluation	n	%
CR	0	0
PR	17	85.0%
SD	3	15.0%
PD	0	0
ORR (95% CI)	17	85.0% (95% CI: 62.1–96.8%)
DCR (95% CI)	20	100.0% (95% CI: 83.2–100.0%)

*CI* confidence interval, *CR* complete response, *DCR* disease control rate, *ORR* overall response rate, *PD* progressive disease, *PR* partial response, *SD* stable disease

One patient achieved a CR after the primary analysis by May 1, 2019. This patient began the study treatment on October 12, 2018 and completed 15 cycles of treatment before CR.

The median PFS time was 7.5 months (95% CI: 6.2–9.4) and the 6 month PFS rate was 88.0%. Median OS was not reached and the 6-month and 12-month OS rates were 100.0 and 68.0%, respectively (Fig. 2c, d).

### Tumor mutation burden

Valid results were obtained from 20 patients. The median TMB value was 1.77 Mb. The ORR was 100.0% (95% CI: 69.2–100.0%) in 10 patients with H-TMB, and 70.0% (95% CI: 34.8–93.3%) in patients with L-TMB. No significant difference in clinical responses were found between H-TMB and L-TMB patients (*P* = 0.211) (Fig. 2e).

## Discussion

In the present study, the results from the G/GEJ adenocarcinoma cohort in a Phase Ib study demonstrated manageable safety and favorable anti-tumor activity of sintilimab combined with a CapeOx regimen as first-line treatment for unresectable advanced metastatic G/GEJ adenocarcinoma.

In terms of safety, the incidence and severity of TRAEs with sintilimab and CapeOx were generally consistent with those of known toxic effects of conventional chemotherapy [26–28] and previously reported side effects of other anti-PD-1 antibody combined with chemotherapy regimens [29, 30]. Platelet count, white blood cell count and neutrophil count decreases were most commonly and mostly grade 1 to 2 reported TRAEs and are expected AEs associated with CapeOx [26–28]. Only 1 patient reported discontinuation of investigational drug application due to a TRAE (abnormal hepatic function). No treatment-related death occurred in this study and in general, the addition of sintilimab to CapeOx showed a manageable safety profile and did not bear extra safety risks.

In the present study, after treatment with sintilimab plus CapeOx, patients with unresectable G/GEJ adenocarcinoma obtained an ORR of 85.0% (95% CI: 62.1–96.8%), which is higher than that of conventional first line chemotherapy. For G/GEJ adenocarcinoma, first-line treatment mainly involves platinum-based chemotherapy and fluoropyrimidine [25]. The ORR of capecitabine-based or oxaliplatin-based therapies for G/GEJ adenocarcinoma was about 30–40% [27, 28]. The ORR for anti-PD-1 antibodies with a chemotherapy regimen were variable. In the KEYNOTE-059 study, the ORR was 60.0% (95% CI: 39.0–79.0%) for pembrolizumab plus cisplatin/5-fluorouracilm (5-FU) as first-line treatment [29]. In the KEYNOTE-062 study, ORRs were 48.6 and 52.5% in patients with a ≥ 1 and ≥ 10 combined positive score (CPS), respectively, after they received pembrolizumab plus cisplatin/5-FU or capecitabine regimen as first-line therapy [31]. In ATTRACTION-04, the ORR for nivolumab with S-1/oxaliplatin was 57.1% (95% CI: 34.0–78.2%) and the ORR for nivolumab with CapeOx was 76.5% (95% CI: 50.1–93.2%) [30]. In another study, the ORR was reported to be 66.7% for an anti-PD1 antibody toripalimab plus CapeOx treatment [32].

Sintilimab plus CapeOx also showed favorable long-term efficacy. Median PFS was 7.5 months (95% CI: 6.2–9.4) and the 6-month PFS rate was 88%. Median OS was not reached and the 6-month and 12- month OS rates were 100.0 and 68.0% respectively, which was higher than for conventional treatments with a median PFS of 5.6 months (95% CI: 5.1–5.7) for capecitabine-cisplatin regimen [27] and a median OS of 11.3 months (95% CI 9.6–13.0) for an epirubicin-oxaliplatin-capecitabine regimen [28]. The median PFS times for anti-PD-1 antibodies with a chemotherapy regimen were variable ranging from 5.7 to 10.6 months, a finding which might be associated with different populations and disease status [30–32].

Next-generation sequencing (NGS) has researcher enabled to perform target capture sequencing, which has been proposed as a reliable technique to identify mutated driver genes and for the estimation of TMBs. Its use has led to the detection of actionable alterations in various cancer related genes [33]. Regarding high TMB and the efficacy of PD-1 treatments, inconsistent results have been reported in previous studies. Wang et al. (2018) suggested that TMB might be associated with better efficacy for PD-1 monotherapy [32], whereas Mishima et al. [34] did not find a significant relationship between TMB and the response of gastric cancers to PD-1 therapy [34]. The latter data is in accordance with our finding that after treatment with sintilimab in combination with CapeOx, no significant difference in the clinical responses was found between H-TMB and L-TMB patients. However, using the median TMB as a cut-off is difficult to extrapolate to the real world clinic and bias due to the small sample size could not be excluded in the present study. In addition, it has been noted that up to now there is no uniform standard for H-TMB [33] and further investigations are urgently required.

## Conclusions

Our results strongly indicate that sintilimab combined with CapeOx is an option for the first-line treatment of patients with advanced or metastatic G/GEJ adenocarcinoma. However, the sample size was small and it was a single-arm study without a comparator. The large scale, double-blinded and randomized Phase III clinical trial ORIENT-16 for previously untreated advanced G/GEJ adenocarcinoma patients is being conducted to evaluate the efficacy and safety of sintilimab combined with CapeOx vs CapeOx alone (ClinicalTrials.gov Identifier: NCT03745170).

## Supplementary information

**Additional file 1: Table S1.** TNM stages of each patient. **Table S2.** Sintilimab related adverse events. **Table S3.** Chemotherapy-related adverse events. **Appendix 1**. Inclusion and exclusion criteria.

## Data Availability

The datasets generated and/or analyzed during the current study are not publicly available since the new drug is being submitted to the National Medical Products Administration for approval, but are available from the corresponding author on reasonable request.

## Abbreviations

AEs: Adverse events
CapeOx: Oxaliplatin/capecitabine
CPS: Combined positive score
CR: Complete response
CT: Computed tomography
DCR: Disease control rate
DOR: Duration of response
ECOG-PS: Eastern Tumor Collaborative Group Performance Status
G: Gastric
GC: Gastric cancer
GEJ: Gastroesophageal junction
HER2: Human epidermal growth factor receptor-2
H-TMB: High tumor mutation burden
L-TMB: Low tumor mutation burden
Mb: Megabase
MRI: Magnetic resonance imaging
NGS: Next-generation sequencing
ORR: Objective response rate
OS: Overall survival
PD: Progressive disease
PD-1: Programmed death-1
PD-L1: Protein programmed death-ligand 1
PFS: Progression-free survival
PR: Partial response
SD: Stable disease
TMB: Tumor mutation burden
TNM: Tumor, nodes and metastases
TRAEs: Treatment-related AEs
TTR: Time to response
UICC: Union for International Cancer Control.
