# Supplementary material for: Safety and efficacy of sintilimab combined with oxaliplatin/capecitabine as first-line treatment in patients with locally advanced or metastatic gastric/gastroesophageal junction adenocarcinoma in a phase Ib clinical trial
Source: BMC Cancer. 2020 Aug 14;20:760. doi: 10.1186/s12885-020-07251-z (PMC7427727; doi:10.1186/s12885-020-07251-z)
Supplement: Supplementary file 1 — Additional file 1: Table S1. TNM stages of each patient. Table S2. Sintilimab related adverse events. Table S3. Chemotherapy-related adverse events. Appendix 1. Inclusion and exclusion criteria. [file 12885_2020_7251_MOESM1_ESM.docx]

# Safety and efficacy of sintilimab combined with oxaliplatin/capecitabine as first- line treatment in patients with locally advanced or metastatic gastric/gastroesophageal junction adenocarcinoma in a phase Ib clinical trial

Haiping Jiang1, Yulong Zheng1, Jiong Qian1, Chenyu Mao1, Xin Xu1, Ning Li1, Cheng Xiao1, Huan Wang1, Lisong Teng2, Hui Zhou3, Shuyan Wang3, Donglei Zhu3, Bo Peng4, Lin Shen5, Nong Xu1*

1 Department of Medical Oncology, The First Affiliated Hospital, School of Medicine, Zhejiang University, Hangzhou, China

2 Department of Surgical Oncology, The First Affiliated Hospital, School of Medicine,

Zhejiang University, Hangzhou, China

3 Department of Medical Science and Strategy Oncology, Innovent Biologics, Inc, Suzhou, China

4 Department of Translational Medicine, Innovent Biologics, Inc, Suzhou, China

5 Department of Medical Oncology, Beijing Cancer Hospital, Beijing, China

# * Corresponding author:

Prof. Nong Xu

Department of Medical Oncology, The First Affiliated Hospital, School of Medicine, Zhejiang University, No 79 Qingchun Road, Hangzhou 310003, China, 86

Tel: +86-13515715262

Fax: +86-0571-87235896

Email: [Nongxu.zhjph@outlook.com](mailto:Nongxu.zhjph@outlook.com)

# Supplementary Table 1. TNM stages of each patient

| **Patient number** | **T** | **N** | **M** |
| --- | --- | --- | --- |
| No. 1 | 4a | 3 | 0 |
| No. 2 | x | x | 1 |
| No. 3 | 4b | 1 | 1 |
| No. 4 | 4a | 2 | 1 |
| No. 5 | x | x | 1 |
| No. 6 | x | x | 1 |
| No. 7 | x | x | 1 |
| No. 8 | 4a | x | 1 |
| No. 9 | 4a | 3 | 0 |
| No. 10 | 4a | x | 1 |
| No. 11 | 4a | 3 | 1 |
| No. 12 | 3 | 3 | 1 |
| No. 13 | 4a | x | 1 |
| No. 14 | x | x | 1 |
| No. 15 | x | x | 1 |
| No. 16 | 4a | 3 | 0 |
| No. 17 | 4a | 3 | 0 |
| No. 18 | 3 | 2 | 1 |
| No. 19 | 4 | 2 | 1 |
| No. 20 | 3 | x | 1 |

T, tumor; N, lymph node; M, metastasis.

# Supplementary Table 2. Sintilimab-related adverse events

|  | All grade n (%) | Grades 3 – 4 n (%) |
| --- | --- | --- |
| **Sintilimab related AEs (n)** | **17 (85.0)** | **5 (25.0)** |
| Platelet count decreased | 7 (35.0) | 3 (15.0) |
| Hypothyroidism | 6 (30.0) | 0 (0.0) |
| White blood cell count decreased | 5 (25.0) | 0 (0.0) |
| Neutrophil count decreased | 4 (20.0) | 0 (0.0) |
| Anaemia | 3 (15.0) | 0 (0.0) |
| Alanine aminotransferase increased | 3 (15.0) | 0 (0.0) |
| Aspartic aminotransferase increased | 2 (10.0) | 0 (0.0) |
| Thyroid function test abnormal | 2 (10.0) | 0 (0.0) |
| Vomiting | 2 (10.0) | 0 (0.0) |
| Hypokalemia | 2 (10.0) | 1 (5.0) |
| Hepatic function abnormal | 2 (10.0) | 1 (5.0) |
| Rash | 2 (10.0) | 0 (0.0) |
| Proteinuria | 2 (10.0) | 0 (0.0) |
| Diarrhea | 1 (5.0) | 1 (5.0) |
| Pneumonitis | 1 (5.0) | 1 (5.0) |

Note: Listed are any grade sintilimab related AEs in ≥ 10% patients and all grade 3 –

4 sintilimab related AEs. AE, adverse events.

# Supplementary Table 3. Chemotherapy-related adverse events

|  | All grade n (%) | Grade 3-4 n (%) |
| --- | --- | --- |
| **Chemotherapy related AEs** | **20 (100.0)** | **11 (55.0)** |
| Platelet count decreased | 15 (75.0) | 9 (45.0) |
| Neutrophil count decreased | 10 (50.0) | 2 (10.0) |
| White blood cell count decreased | 9 (45.0) | 0 (0.0) |
| Alanine aminotransferase increased | 4 (20.0) | 0 (0.0) |
| Rash | 4 (20.0) | 0 (0.0) |
| Vomiting | 3 (15.0) | 0 (0.0) |
| Hepatic function abnormal | 3 (15.0) | 1 (5.0) |
| Anaemia | 3 (15.0) | 0 (0.0) |
| Nausea | 2 (10.0) | 0 (0.0) |
| Hyperchlorhydria | 2 (10.0) | 0 (0.0) |
| Hypaesthesia | 2 (10.0) | 0 (0.0) |
| Hypokalemia | 2 (10.0) | 1 (5.0) |
| Pyrexia | 2 (10.0) | 0 (0.0) |
| Proteinuria | 2 (10.0) | 0 (0.0) |
| Aspartic aminotransferase increased | 2 (10.0) | 0 (0.0) |
| γ-glutamyl transferase increased | 1 (5.0) | 1 (5.0) |
| Diarrhea | 1 (5.0) | 1 (5.0) |

Note: Listed are any grade chemotherapy related AEs in ≥ 10% patients and all grade 3 – 4 chemotherapy related AEs. AE, adverse events.

# Appendix 1

## Inclusion criteria

1. Inoperable, locally advanced recurrent or metastatic adenocarcinoma of the gastric/gastroesophageal junction confirmed by histopathological examination.
2. Exclusion of patients with known HER2 gene amplification or overexpression.
3. Patient had not received chemotherapy for advanced disease or had disease progression after finishing systemic adjuvant therapy for > 6 months.
4. Signed written informed consent and were able to follow protocol visits and related procedures.
5. Aged from 18 to 70 years.
6. Expected survival time > 12 weeks.
7. Had at least one measurable or evaluable lesion per Response Evaluation Criteria in Solid Tumors (RECIST ver. 1.1).
8. Had an Eastern Cooperative Oncology Group performance (ECOG) status of 0 or 1.
9. Female subjects of reproductive age or male subjects with a spouse at a reproductive age were required to take effective birth control during the whole treatment period and for 6 months afterwards.
10. Had sufficient organ and bone marrow functions defined as follows:

1) Blood routine: ANC ≥ 1.5 × 109/L; PLT ≥ 100 × 109/L; HGB ≥ 9.0 g/dL.

- 1. Liver Function: TBIL ≤ 1.5 × ULN; for those patients with HCC, hepatic metastases or Gilbert syndrome or suspect cases (persistent or recurrent hyperbilirubinemia is mainly unbound bilirubin without the proof of hemolysis or hepatopathy), TBIL ≤ 3 × ULN; for those patients without HCC and hepatic metastases, ALT and AST ≤ 2.5 × ULN; for those patients with

HCC or hepatic metastases, ALT or AST ≤ 5 × ULN.

- 1. Renal function: Cr ≤ 1.5 × ULN or CCr ≥ 50 mL/min; urine routine results measured protein < 2+; for those patients with PRO ≥ 2+ in urinalysis at baseline, urine was collected over 24 h and the protein in urine should be < 1 g in 24 h.
  2. Coagulation function: APTT and INR ≤ 1.5 × ULN.
  3. TSH or FT4 was within the normal range.

## Exclusion criteria

1. Previously treated with PD-1/PD-L1 antibodies.
2. Had a history of any Common Terminology Criteria Adverse Event (CTCAE) ≥ III irAE when receiving any immunotherapy drug. Previously treated with ipilimumab, unless all of the following criteria were met:
   1. Ipilimumab-related AEs were completely cured and the treatment of irAE completed 4 weeks before the first dose of the study drug was administered.
   2. The first dose of ipilimumab was taken at least 12 weeks before the first dose of the study drug and the last dose of ipilimumab was given at least 6 weeks before the first dose of the study drug.
   3. Grade 4 irAE was not observed with ipilimumab or grade 3 irAE that required treatment for more than 4 weeks.
   4. Patient had obvious progressive disease after the last administered dose of ipilimumab.
3. Involved in another interventional clinical study (unless participating in an observational (non-interventional) clinical study or at the follow-up stage of an interventional study).
4. Received any investigational drugs within 4 weeks prior to the first dose of the

study drug.

1. Received a final dose of antitumor therapy (chemotherapy, endocrine therapy, targeted therapy, immunotherapy or tumor embolization, etc.) within 3 weeks of the first dose of the study therapy, received a final dose of biological products, nitroso urea or mitomycin C treatment (if the biological product also belongs to the class of anti-tumor endocrine therapy, refer to the above mentioned exclusion criteria) within 6 weeks of the first dose of the study therapy.
2. Immunosuppressive drugs were used within 4 weeks prior to the first dose of the study treatment excluding nasal sprays, inhalation or another routes of local glucocorticoids or systemic glucocorticoids at a physiological dose (i.e., no more than 10 mg/day of prednisone or an equivalent dose of other glucocorticoids).
3. Live attenuated vaccine was given within 4 weeks prior to the first dose of the study treatment or was planned to be administered during the study period.
4. Had undergone major surgery (craniotomy, thoracotomy, or laparotomy) or unhealed wounds, ulcers or fractures within 4 weeks prior to the first dose of treatment.
5. Toxicity not recovered to NCI CTCAE ver. 4.03 grade 0 or grade 1 (excluding hair loss) due to previous antitumor therapy prior to the first dose of the study therapy.
6. Previously received total pelvic radiotherapy.
7. Known patients with meningeal metastasis; other known patients with uncontrolled or untreated central nervous system metastases, excluding patients who had received treatment with stable symptoms and stopped glucocorticoid and anticonvulsant therapy more than 4 weeks before the first dose of the study therapy.
8. Active, known autoimmune disease or a 2-year history of the disease (for patients

with vitiligo, psoriasis, alopecia or Graves' disease who did not require systemic treatment within the past 2 years, hypothyroidism only requiring thyroid hormone replacement therapy and type 1 diabetes only requiring insulin replacement therapy could be enrolled). Patients with positive autoimmune antibodies should be assessed by the investigator to confirm the existence of autoimmune disease.

1. Known history of primary immunodeficiency.
2. Known history of tuberculosis.
3. Known history of allogeneic organ transplantation and allogeneic hematopoietic stem cell transplantation.
4. Known allergy to any ingredient of sintilimab; the patient has a history of severe allergic reactions to other monoclonal antibodies; the patient was previously allergic to capecitabine or oxaliplatin.
5. Uncontrolled concurrent diseases including but not limited to:

- HIV infected patient (HIV 1/2 antibody positive)
- A severe infection that is active or not under clinical control
- Symptomatic congestive heart failure (New York heart association, grade II-

IV) or symptomatic or poorly controlled arrhythmias

- Arterial hypertension (systolic blood pressure 160 mmHg or diastolic blood pressure 100 mmHg) that is still uncontrolled even after standard treatment
- Any arterial thromboembolic events, including myocardial infarction, unstable angina, cerebrovascular accident, or transient ischemic attack that occurred within 6 months prior to enrollment for treatment
- Required immediate intervention of esophageal or gastric varices (binding or hardening treatment) or according to the researchers' opinions or gastroenterology and hepatology expert opinions believe the bleeding risk is

high; evidence of portal hypertension (imaging examination showed splenomegaly) or a history of varices bleeding; patients must undergo endoscopic evaluation within 3 months before enrollment

- Any life-threatening bleeding or grade 3 or 4 gastrointestinal/varicose bleeding requiring a blood transfusion, endoscopy, or surgery occurred within 3 months prior to enrollment
- A history of deep venous thrombosis, pulmonary embolism, or any other serious thromboembolism (implantable port of intravenous infusion or catheter-derived thrombosis, or superficial venous thrombosis is not considered as "severe" thromboembolism) within 3 months prior to enrollment
- Uncontrolled metabolic disorders or other non-malignant organs or systemic diseases or secondary reactions to cancer which may lead to higher medical risks and/or uncertainty in the evaluation of survival
- Hepatic encephalopathy, hepatorenal syndrome or Child-PUGH grade B or more severe cirrhosis
- History of ileus or inflammatory bowel disease or extensive intestinal resection (partial colectomy or extensive intestinal resection with chronic diarrhea) or Crohn's disease, ulcerative colitis or chronic diarrhea
- Other acute or chronic illness, mental illness, or abnormal laboratory values may lead to following results: Increased risk because of study participation or study drug administration, interferes with the interpretation of the findings, or according to the judgment of the researcher, the patient was classified as not eligible to participate in this study.

1. Patients with acute or chronic active hepatitis B or C (for patients with sintilimab monotherapy, if HBsAg is positive but HBV DNA copy number < 1 × 104/mL they

could be enrolled; for patients treated with sintilimab plus chemotherapy, if HBsAg was positive but HBV DNA lower than the detection limit they could be enrolled; prevention and treatment requirements refer to the 2015 edition of the chronic hepatitis B prevention guidelines; patients with hepatitis C in the inactive phase could be enrolled).

1. History of gastrointestinal perforation and/or fistula within 6 months prior to enrollment.
2. Interstitial lung disease (including past and present).
3. Clinically uncontrollable third interstitial effusion, such as pleural effusion and ascites that cannot be controlled by drainage or other methods before enrollment.
4. History of other primary malignancies, excluding:

- Cured malignancies; had no known active disease for more than 5 years prior to enrollment and had a very low risk of recurrence
- Non-melanoma skin cancer or malignant freckle mole with adequate treatment and no evidence of disease recurrence
- Carcinoma *in situ* with adequate treatment and no evidence of recurrence

1. Women who were pregnant or breastfeeding.
